# 691. Epidemiology of Viral Respiratory Infections in Pediatric Transplant: Initial results from the Viral Infections in Pediatric Transplant Recipients (VIPER) Study

**DOI:** 10.1093/ofid/ofaf695.230

**Published:** 2026-01-11

**Authors:** Gabriela Maron, Sarah E Longserre, Jose Ferrolino, Ronald H Dallas, Madeline Johnson, Sarah K Johnson, Alexander L Greninger, Margaret Mills, Lara A Danziger-Isakov, Jason B Weinberg, Maria A Garcia Fernandez, Jonathan Albert, Elizabeth A Moulton, Maria S Rueda-Altez, Tanvi S Sharma, Timothy D Minniear, Benjamin Hanisch, Alpana Waghmare, Anna R Huppler, Marc Foca, Beth K Thielen, Madan Kumar, Natalie J Dailey Garnes, Kari Neemann, Mary Suzanne S Whitworth, Kathryn P Goggin, Carol Kao, Ivan A Gonzalez, Masako Shimamura, ramia g zakhour, Lauren Powell, Harbir Arora, Victoria A Statler, Elaine Tuomanen, Betsy Herold, William Steinbach, Janet A Englund

**Affiliations:** St. Jude Children's Research Hospital, Memphis, Tennessee; St. Jude Children's Research Hospital, Memphis, Tennessee; St . Jude Children’s Research Hospital, Memphis, Tennessee; St. Jude Children's Research Hospital, Memphis, Tennessee; St. Jude Children's Research Hospital, Memphis, Tennessee; UAMS, Little Rock, Arkansas; University of Washington, Seattle, Washington; University of Washington, Seattle, Washington; Cincinnati Children's Hospital, Cincinnati, OH; University of Michigan, Ann Arbor, Michigan; Arkansas Children’s Hospital, Little Rock, Arkansas; UPMC Childrens Hospital of Pittsburgh, Pittsburgh, PA; Baylor College of Medicine/Texas Children's Hospital, Houston, Texas; The University of Alabama at Birmingham; Boston Children's Hospital, Boston, MA; University of Tennessee Health Science Center, Memphis, Tennessee; Children’s National Hospital, Washington, DC, USA, Washington, District of Columbia; Seattle Children's Hospital/Fred Hutchinson Cancer Center, Seattle, Washington; Medical College of Wisconsin, Wauwatosa, Wisconsin; Albert Einstein College of Medicine, Jersey City, New Jersey; University of Minnesota, Minneapolis, Minnesota; University of Chicago, Chicago, IL; The University of Texas MD Anderson Cancer Center; University of Nebraska Medical Center - Children's Nebraska, Omaha, Nebraska; Cook Children's Health Care System, Fort Worth, Texas; Emory University School of Medicine and Children's Healthcare of Atlanta, Atlanta, Georgia; WASHINGTON UNIVERSITY IN ST LOUIS SCHOOL OF MEDICINE, ST LOUIS, Missouri; University of Miami Miller School of Medicine, Miami, Florida; Nationwide Children's Hospital, Columbus, Ohio; University of Texas at Houston, houston, Texas; Medical University of South Carolina, Charleston, South Carolina; Central University of Michigan School of Medicine, Detroit, Michigan; Norton Children's and University of Louisville School of Medicine, Louisville, KY; St Jude Children's Research Hospital, Memphis, Tennessee; Albert Einstein College of Medicine, Jersey City, New Jersey; Arkansas Children's, University of Arkansas for Medical Sciences, Little Rock, AR; Seattle Children’s Hospital/Univ. Washington, Seattle, Washington

## Abstract

**Background:**

Respiratory viral infections (RVIs) cause substantial morbidity in pediatric transplant recipients. Previous efforts to establish the epidemiology of RVIs are mainly retrospective, with limited evaluation of association between pre-transplant screening of respiratory viruses and future RVIs. The VIPER study is a prospective, multicenter cohort study investigating the epidemiology and impact of RVIs in these patients.

**Figure:**
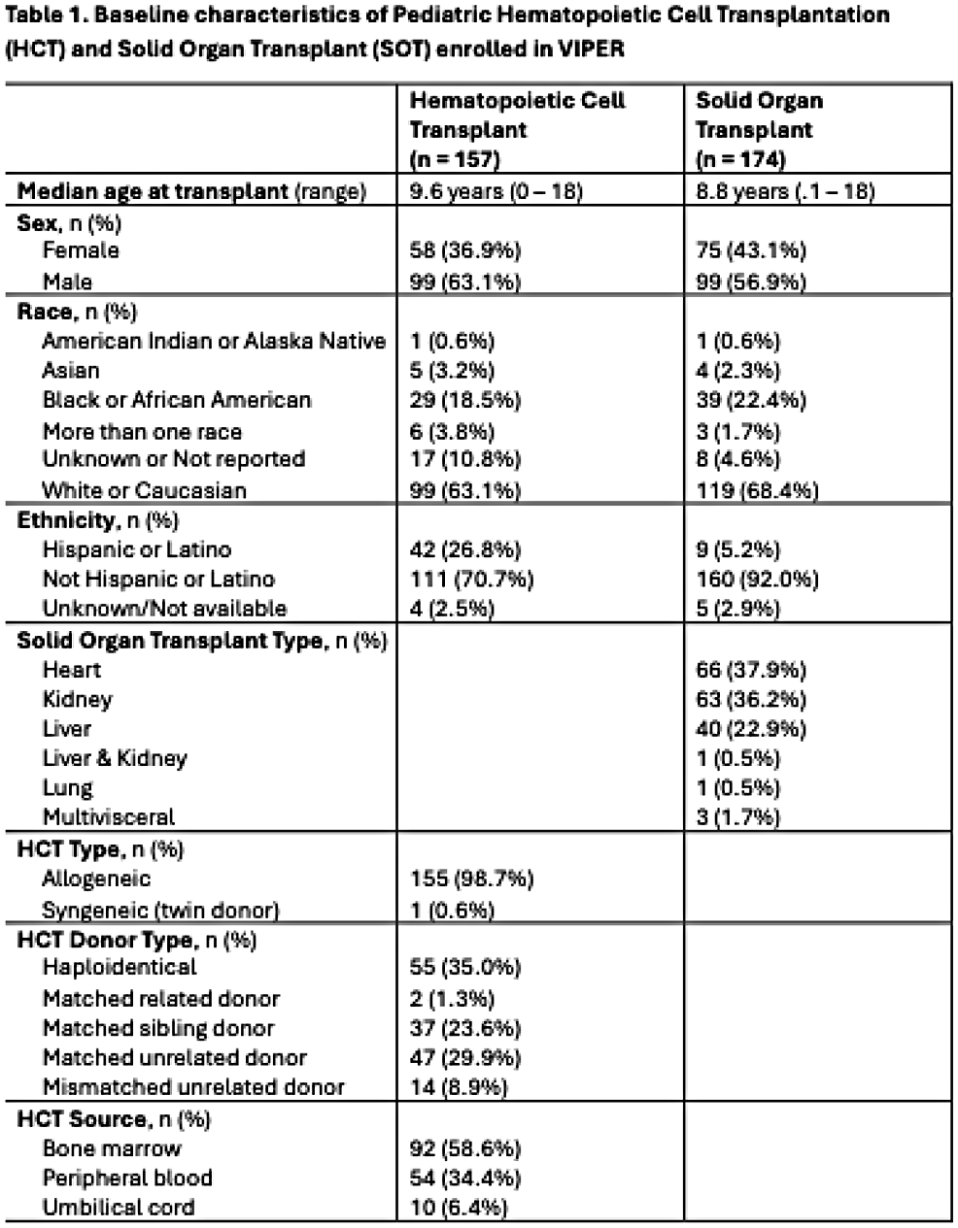

**Figure 1:**
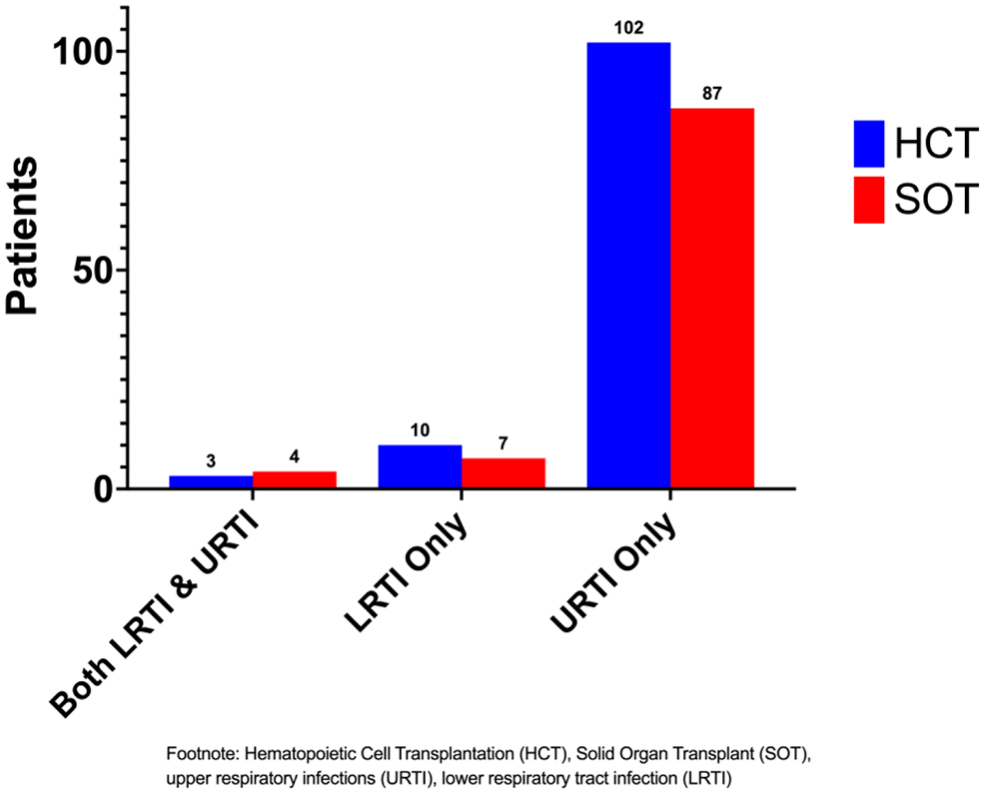
Clinical Presentation of Respiratory Viral Infections Post Transplant

**Methods:**

Patients undergoing Hematopoietic Cell Transplantation (HCT) and Solid Organ Transplant (SOT) at 26 US institutions were enrolled at transplant and monitored for RVIs for a year. Nasal swabs (NS) were collected at baseline and first clinical RVI, and clinical data extracted from medical records. Baseline NS were tested for viruses using standard real time PCR methods (OpenArray, ThermoFischer Scientific) and compared with clinical PCR results at first RVI. We summarize the preliminary clinical data and analyze the association between baseline testing and the first RVI with univariate logistic regression. The study is conducted through the Pediatric Infectious Diseases Transplant Network (PIDTRAN).

**Figure 2:**
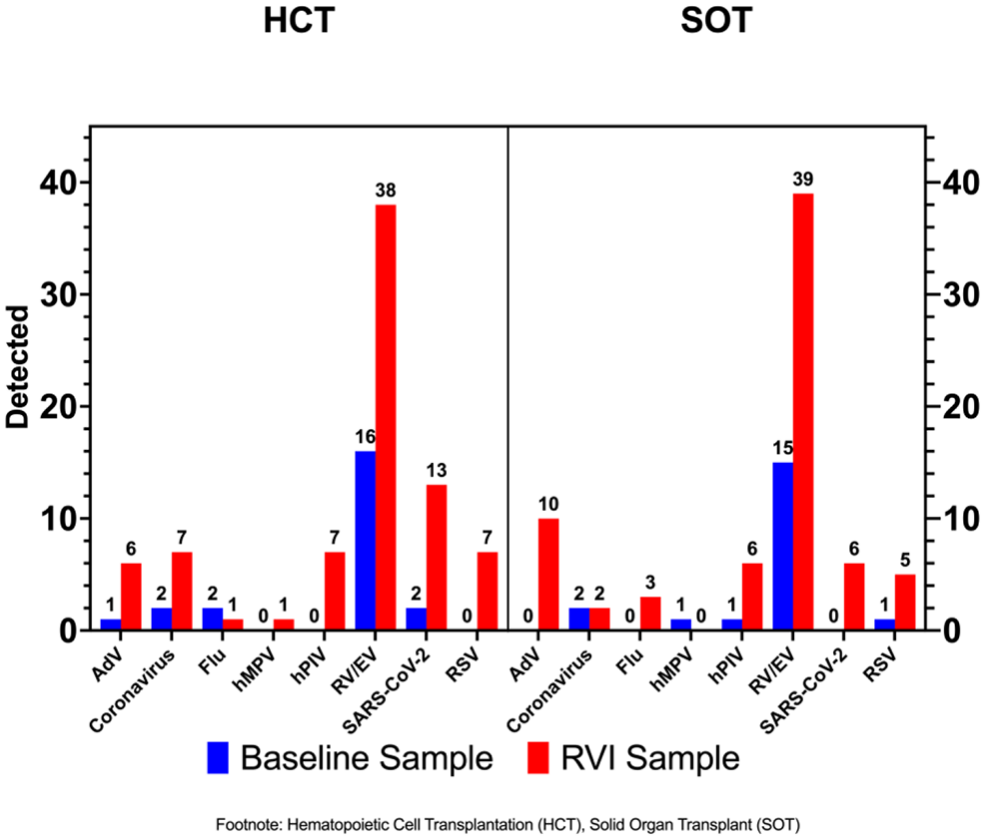
Respiratory Viruses Detected at Baseline and at First Respiratory Viral Infection Episode

**Results:**

157 HCT and 174 SOT recipients were included. Demographic and transplant characteristics are described in Table 1. HCT patients had more RVIs vs SOT (77% vs 69%). Median time from transplant to first RVI was 99 days for HCT and 112 days for SOT. Upper respiratory tract infections (URTI) were common in both groups (Figure 1) but more SOT than HCT recipients progressed to lower respiratory tract infection (LRTI). Rhinovirus/enterovirus (RhV/EV) were the most frequent viruses in HCT and SOT, both at baseline and first RVI (Figure 2). Among LRTI, the most frequent viruses were RhV/EV (48%), Adenovirus (16%) and RSV (13%). Only baseline RhV/EV predicted RhV/EV at first RVI (OR:2.9, CI:1.2-6.9, p=0.02).

**Conclusion:**

Many transplant recipients carry respiratory viruses at the time of transplant. Most post- transplant RVIs were URTI. RhV/EV were the most frequent virus detected overall, including in LRTI. RhV/EV at baseline predicted subsequent RhV/EV RVI. No other virus demonstrated this association. Analysis is limited by the current sample size, but the continuing accrual will allow for a more robust analysis.

**Disclosures:**

Lara A. Danziger-Isakov, MD, MPH, AiCuris.: Grant/Research Support|Ansun BioPharma: Grant/Research Support|Astellas Pharma Global Development, Inc: Advisor/Consultant|Astellas Pharma Global Development, Inc: Grant/Research Support|kamada: Advisor/Consultant|Merck Sharp & Dohme Corporation: Grant/Research Support|Pfizer (Any division): Grant/Research Support|Takeda Pharmaceuticals: Grant/Research Support

